# A practical approach to estimating optic disc dose and macula dose without treatment planning in ocular brachytherapy using ^125^I COMS plaques

**DOI:** 10.1186/s13014-018-1166-z

**Published:** 2018-11-13

**Authors:** Yongsook C. Lee, Shih-Chi Lin, Yongbok Kim

**Affiliations:** Department of Radiation Oncology, The University of Arizona, 3838 N. Campbell Avenue, Building #2, Tucson, AZ 85719 USA

**Keywords:** Ocular brachytherapy, ^125^I, COMS plaques, Optic disc dose, Macula dose

## Abstract

**Background:**

It has been reported that proximity of the tumor to the optic disc and macula, and radiation dose to the critical structures are substantial risk factors for vision loss following plaque brachytherapy. However, there is little dosimetry data published on this. In this study, therefore, the relationship between distance from tumor margin and radiation dose to the optic disc and macula in ocular brachytherapy using ^125^I Collaborative Ocular Melanoma Study (COMS) plaques was comprehensively investigated. From the information, this study aimed to allow for estimation of optic disc dose and macula dose without treatment planning.

**Methods:**

An in-house brachytherapy dose calculation program utilizing the American Association of Physicists in Medicine Task Group-43 U1 formalism with a line source approximation in a homogenous water phantom was developed and validated against three commercial treatment planning systems (TPS). Then optic disc dose and macula dose were calculated as a function of distance from tumor margin for various tumor basal dimensions for seven COMS plaques (from 10 mm to 22 mm in 2 mm increments) loaded with commercially available ^125^I seeds models (IAI-125A, 2301 and I25.S16). A prescribed dose of 85 Gy for an irradiation time of 168 h was normalized to a central-axis depth of 5 mm. Dose conversion factors for each seed model were obtained by taking ratios of total reference air kerma per seed at various prescription depths (from 1 mm to 10 mm in 1 mm intervals) to that at 5 mm.

**Results:**

The in-house program demonstrated relatively similar accuracy to commercial TPS. Optic disc dose and macula dose decreased as distance from tumor margin and tumor basal dimension increased. Dose conversion factors increased with increasing prescription depth. There existed dose variations (<8%) among three ^125^I seed models. Optic disc dose and macula dose for each COMS plaque and for each seed model are presented in a figure format. Dose conversion factors for each seed model are presented in a tabular format.

**Conclusions:**

The data provided in this study would enable clinicians in any clinic using ^125^I COMS plaques to estimate optic disc dose and macula dose without dose calculations.

**Electronic supplementary material:**

The online version of this article (10.1186/s13014-018-1166-z) contains supplementary material, which is available to authorized users.

## Background

Plaque brachytherapy is currently the most common treatment option for early stage or medium-sized intraocular tumors (≤10 mm in apical height and ≤ 16 mm in diameter for uveal melanomas) [1–3]. It offers equivalent tumor control and better quality of life such as eye preservation and vision retention in comparison to enucleation [3–5]. Various plaque designs were proposed and are clinically used in major institutions [6–8]. Nonetheless, Collaborative Ocular Melanoma Study (COMS) plaques have been widely used in most clinics since the COMS established standardized methods of plaque brachytherapy for medium-sized choroidal melanomas [3].

In plaque brachytherapy for intraocular tumors, major critical structures related to vision are lens, optic nerve (optic disc) and macula (fovea). A cataract, clouding of the lens, is the most common radiotherapy contraindication but a surgery can restore vision loss due to cataracts. On the other hand, radiation damage to the optic disc and macula can cause permanent vision loss which is usually not recoverable. Several studies reported outcomes for vision deterioration/loss following plaque brachytherapy [4, 5, 9–13]. Some of the studies revealed that proximity of the tumor to the optic disc and fovea, and radiation dose are substantial risk factors for vision loss [4, 5, 9]. However, there is a paucity of literature on the relationship between proximity of the tumor to the vision-related critical structures and radiation dose to them in plaque brachytherapy.

Therefore, this study has comprehensively examined the relationship between distance from tumor margin and radiation dose to the optic disc or macula in ocular brachytherapy using ^125^I COMS plaques through a dosimetry study. By providing the dosimetry data, this study aims to enable clinicians (both ophthalmologist and radiation oncologist) in any clinic or institution using ^125^I COMS plaques to predict optic disc dose and macula dose at the time of tumor size measurements without dose calculations in a treatment planning system (TPS).

The American Association of Physicists in Medicine (AAPM) Task Group (TG) 129 recommends that in dose calculations, heterogeneity corrections be accounted for non-tissue materials such as gold-alloy backing and silastic seed carrier insert in the plaque [3]. As of today, however, there is no commercially available TPS taking into account heterogeneity corrections. Furthermore, the hybrid method, homogeneous dose calculations multiplied by known heterogeneity correction factors, suggested by the AAPM TG 129, is limited to the obsolete ^125^I seed model 6711 [3] and there is no correction factor provided for currently available ^125^I seed models. Herein, in current clinical practice, the AAPM TG-43 dosimetry formalism with a line source approximation in a homogeneous water medium is widely used. In this study, dose calculations were performed based on the current clinical practice.

## Methods

### Determination of parameters required for treatment planning

**Fig. 1:**
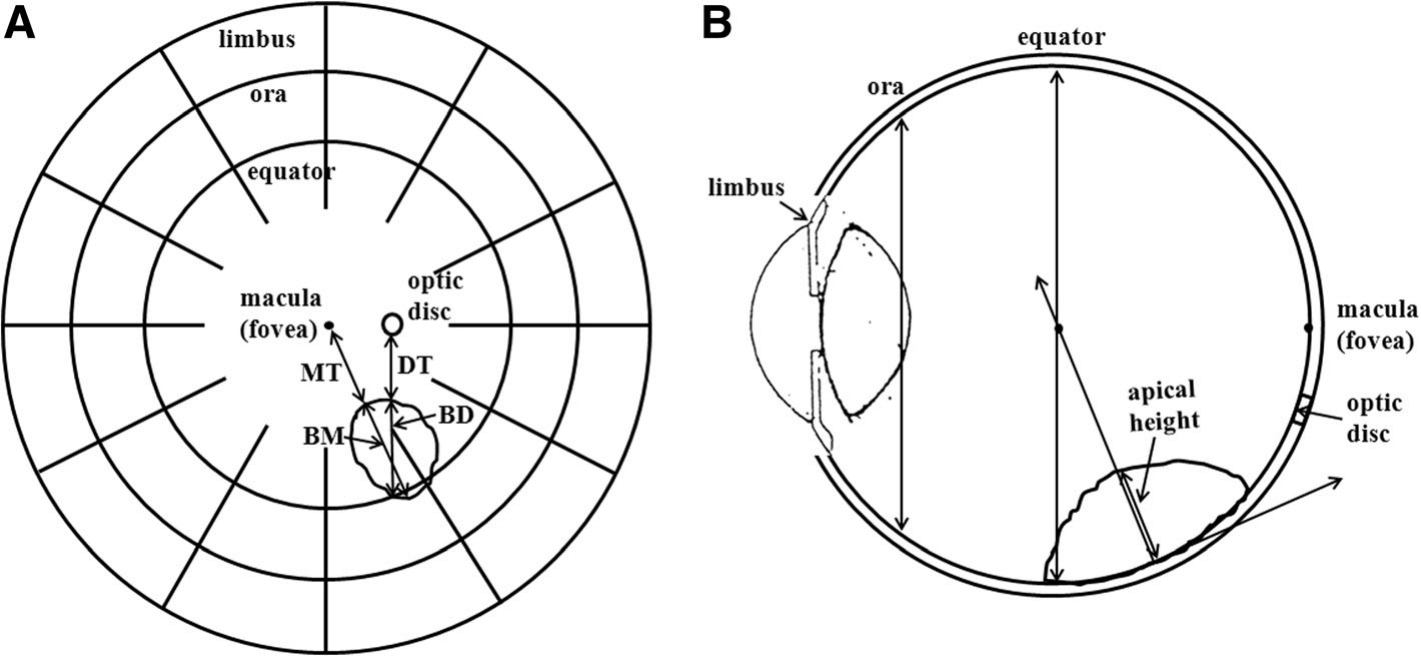
**a** The fundus diagram illustrating BD, DT, BM and MT of the tumor and **b** the cross section diagram of the eye showing apical height of the tumor

Following COMS protocols [14], five parameters required for treatment planning were determined in an ophthalmologist’s office. Tumor basal dimension at center in the direction from optic disc (BD, parameter #1) and distance from optic disc to tumor margin (DT, parameter #2) were measured in a fundus diagram. Tumor basal dimension at center in the direction from macula (BM, parameter #3) and distance from macula to tumor margin (MT, parameter #4) were also measured in the same fundus diagram. Tumor height (parameter #5), which determines a prescription depth, was measured using ultrasound. The fundus diagram in Fig. 1a illustrates BD, DT, BM and MT of the tumor and the cross section diagram of the eye in Fig. 1b shows apical height of the tumor. Adequate plaque size was determined by adding a margin of 2–3 mm to the largest tumor dimension. Then this information was sent to the department of radiation oncology for treatment planning.

### Validation of our in-house brachytherapy dose calculation program

For efficient calculations of optic disc dose and macula dose as a function of parameters mentioned above, an in-house brachytherapy dose calculation program was developed in MATLAB® software (vR2016a, MathWorks, Natick, MA) and validated against three commercial TPS for benchmark calculations in the literature [15]. The conventional AAPM TG-43 Update (TG-43 U1) dosimetry formalism with a line source approximation in a homogeneous water medium was incorporated into the in-house program. Parameters for the TG-43 U1 formalism including radial dose function (Table II in TG-43 U1) and anisotropy function (Table V in TG-43 U1) were taken from the TG-43 U1 [16]. The step size over distance “r” and polar angle “θ” for radial dose function and anisotropy function was coarser in the TG-43 U1 than that in Rivard et al. study. In our in-house program, linear interpolation was used to obtain radial dose function and anisotropy function values, while in Rivard et al., log-linear interpolation was used for radial dose function values and linear interpolation for anisotropy function values. Seed coordinates for COMS plaques taken from Table I in the AAPM TG 129 [3] were also incorporated into the program. In Rivard et al.’s benchmark test, doses at several points along the central-axis and at off-axis points for organs at risk (OARs) were calculated in commercial TPS for a 16 mm COMS plaque loaded with ^125^I seeds (Amersham Oncoseed 6711). The TPS include P^3^ (Pinnacle^3^, v8.0dpl, Philips Medical Systems, Cleveland, OH), BV (BrachyVision™, v8.1, Varian Medical Systems, Inc., Palo Alto, CA) and PS (Plaque Simulator, v5.3.9, Eye Physics LLC, Los Alamitos, CA) which all use a line source approximation in homogeneous water phantoms [15]. In all three TPS, air-kerma strength (S_k_) per seed (unit: U = μGym^2^h^− 1^) was kept the same (4.572 U) to deliver approximately 85 Gy to a central-axis depth of 5 mm for an irradiation time of 100 h. The central-axis depth is the distance along the plaque central-axis from the inner sclera. Central-axis and off-axis point doses for this benchmark test were calculated in our in-house program. Total reference air kerma (TRAK = S_k_ × irradiation time) per seed (unit: μGym^2^) was kept the same (4.572 U × 100 h) as in the benchmark test. Then, for each point, our data were compared with those in Rivard et al.’s study by computing the modulus of a relative percent difference in dose using the following equation:

$$ \left|{\mathrm{D}}_{\mathrm{diff}}^{\mathrm{Rel}}\left(\%\right)\right|=\frac{\left|{\mathrm{D}}_{\mathrm{in}}-{\mathrm{D}}_{\mathrm{ref}}\right|}{{\mathrm{D}}_{\mathrm{ref}}}\times 100 $$

where D_in_ is dose calculated in our in-house program and D_ref_ is reference dose from Rivard et al.’s study.

### Calculations of optic disc dose and macula dose for standard COMS plaques loaded with ^125^I seeds

Optic disc dose and macula dose for standard COMS plaques were calculated in the in-house program. Dose calculations were performed as a function of distance from tumor margin (DT or MT) up to 10 mm for various tumor basal dimensions (BD or BM) (<20 mm in 2 mm intervals). A prescribed dose of 85 Gy for an irradiation time of 168 h was normalized at a central-axis depth of 5 mm. The calculations were performed for all seven different-sized COMS plaques (from 10 mm to 22 mm in diameter in 2 mm increments) and for three currently, commercially available ^125^I seeds models (IsoAid Advantage IAI-125A, Best Industries 2301 and Bebig I25.S16) of the seed models listed in the AAPM TG 129 [3]. Parameters in the dosimetry formalism for the three seed models were taken from the AAPM TG-43 U1 [16] and supplement to the AAPM TG-43 U1 [17].

### Generation of dose conversion factors for different prescription depths

Since a prescription depth is determined based on the tumor apex (COMS protocols [14] or American Brachytherapy Society (ABS) guidelines [18]) and it is not always 5 mm, dose conversion factors for different prescription depths were generated in the in-house program. Optic disc dose and macula dose were calculated for prescription depths from 1 mm to 10 mm in 1 mm intervals. The prescribed dose (85 Gy) and irradiation time (168 h) were kept the same as for prescription depth of 5 mm. Then ratios of TRAK per seed to obtain 85 Gy to each prescription depth to that to 5 mm were taken as dose conversion factors. The calculations were performed for all seven COMS plaques and for the three seed models mentioned above.

## Results

### Validation of our in-house brachytherapy dose calculation program

Table 1 presents the comparison of central-axis dose values for a 16 mm COMS plaque loaded with ^125^I seeds (model 6711) between our in-house program and three TPS used in Rivard et al.’s study [15]. Max$\left|{\mathrm{D}}_{\mathrm{diff}}^{\mathrm{Rel}}\left(\%\right)\right|$ is the largest modulus of relative percent differences in dose between the two studies. Max$\left|{\mathrm{D}}_{\mathrm{diff}}^{\mathrm{Rel}}\left(\%\right)\right|$ ranged from 0.32% to 2.35% and the largest difference (2.35%) occurred at the farthest dose point (22.6 mm, opposite retina).

**Table 1:** The comparison of central-axis dose values (in Gy) for a 16 mm COMS plaque loaded with ^125^I seeds (model 6711) calculated in our in-house program with those in three commercial treatment planning systems (TPS) in Rivard et al.’s study

d (mm)	CAX points	Current Study	Data from Table II in Rivard et al. [15]			Max$\left|{\mathrm{D}}_{\mathrm{diff}}^{\mathrm{Rel}}\left(\%\right)\right|$
			P^3^	BV	PS	
−1.0	Outer sclera	336.18	341	340	339	1.41
0.0	Inner sclera	258.79	261	261	260	0.85
1.0		202.10	203	203	202	0.44
2.0		160.48	161	161	160	0.32
3.0		128.68	129	129	128	0.53
4.0		103.91	104	104	103	0.88
5.0	Rx depth	84.52	84.4	84.5	83.9	0.74
6.0		69.26	69.2	69.2	68.8	0.67
7.0		57.27	57.2	57.2	56.9	0.65
8.0		47.73	47.7	47.7	47.4	0.70
9.0		40.12	40.0	40.0	39.8	0.81
10.0		34.00	33.9	33.9	33.7	0.89
11.3	Eye center	27.73	27.6	27.6	27.5	0.83
15.0		16.45	16.3	16.3	16.3	0.95
20.0		9.02	8.87	8.89	8.84	2.04
22.6	Opposite retina	6.83	6.70	6.70	6.67	2.35

P^3^, BV and PS represent Pinnacle, BrachyVision and Plaque Simulator, respectively. Dose values were calculated for a prescribed dose of approximately 85 Gy to a central-axis depth of 5 mm (S_k_ = 4.752 U and irradiation time = 100 h) using a line source approximation of the AAPM TG-43 formalism and homogeneous water phantoms. Max$\left|{\mathrm{D}}_{\mathrm{diff}}^{\mathrm{Rel}}\left(\%\right)\right|$ is the largest modulus of relative percent differences in dose between the two studies

Table 2 presents the comparison of doses at OAR points (fovea, optic disc center, lens center and lacrimal glad center) for four different plaque positions (#1-#4) between our study and Rivard et al.’s. From Fig. 3 in Rivard et al., the plaque positions #1, #2, #3 and #4 were centered on equator on temporal side (9 o’clock), on nasal side (3 o’clock), on superior side (12 o’clock) and on inferior side (6 o’clock), respectively [15]. Coordinates for the OARs were taken from Rivard et al. [15]. $\left|{\mathrm{D}}_{\mathrm{diff}}^{\mathrm{Rel}}\left(\%\right)\right|$ was defined in the same way as for the central axis dose comparison except that D_ref_ was an average value of off-axis doses calculated from all TPS [15]. Off-axis dose differences between the two studies ranged from 0.40% to 1.52% except for the lacrimal gland point for plaque positions #1 (4.39%) and #3 (4.40%).

**Table 2:** The comparison of dose values (in Gy) at organs at risk points (fovea, optic disc center, lens center and lacrimal glad center) for four different plaque positions (#1-#4) [15] of the 16 mm COMS plaque loaded with ^125^I seeds (model 6711) calculated in our in-house program with average off-axis dose values calculated from treatment planning systems used in Rivard et al.’s study

Plaque position	Off-axis location	Current Study	Data from Table III in Rivard et al. [15]	$\left|{\mathrm{D}}_{\mathrm{diff}}^{\mathrm{Rel}}\left(\%\right)\right|$
#1	Fovea	16.50	16.3	1.25
	Optic disc	11.29	11.2	0.79
	Lens	21.59	21.5	0.40
	Lacrimal gland	40.92	39.2	4.39
#2	Fovea	16.50	16.3	1.25
	Optic disc	28.02	27.6	1.52
	Lens	21.59	21.5	0.40
	Lacrimal gland	7.19	7.1	1.22
#3	Fovea	16.50	16.3	1.25
	Optic disc	16.48	16.3	1.08
	Lens	21.59	21.5	0.40
	Lacrimal gland	40.92	39.2	4.40
#4	Fovea	16.50	16.3	1.25
	Optic disc	16.48	16.3	1.08
	Lens	21.59	21.5	0.40
	Lacrimal gland	7.19	7.1	1.22

Dose values were calculated for a prescribed dose of approximately 85 Gy to a central-axis depth of 5 mm (S_k_ = 4.752 U and irradiation time = 100 h) using a line source approximation of the AAPM TG-43 formalism and homogeneous water phantoms. $\left|{\mathrm{D}}_{\mathrm{diff}}^{\mathrm{Rel}}\left(\%\right)\right|$ is a modulus of a relative percent difference in dose between the two studies

### Optic disc dose and macula dose for standard COMS plaques loaded with ^125^I seeds

**Fig. 2:**
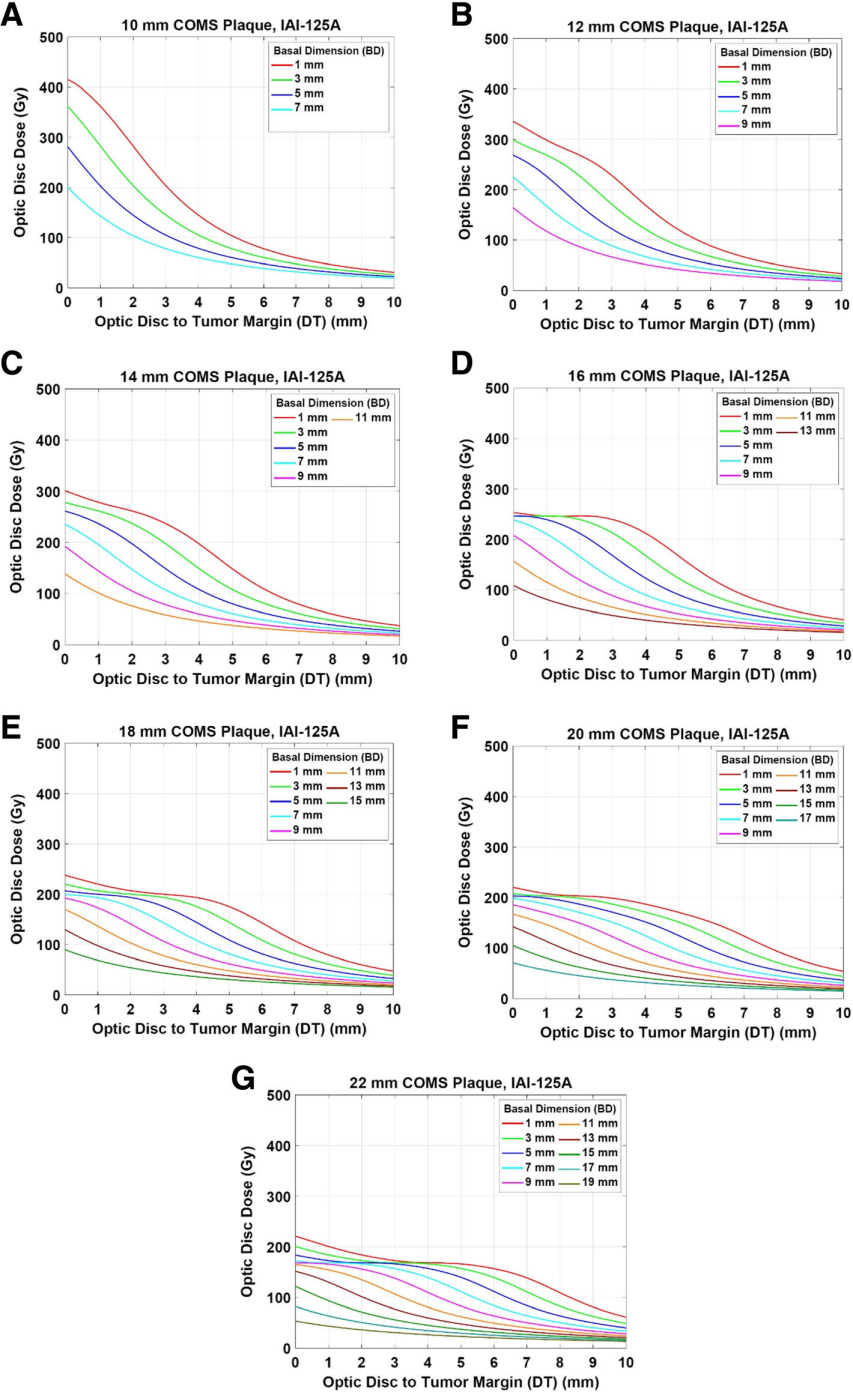
**a**-**g** Optic disc dose as a function of optic disc-to-tumor margin distance (DT) for various tumor basal dimensions (BD) for seven COMS plaques loaded with ^125^I seeds (model IAI-125A) when 85 Gy is prescribed to a central-axis depth of 5 mm

**Fig. 3:**
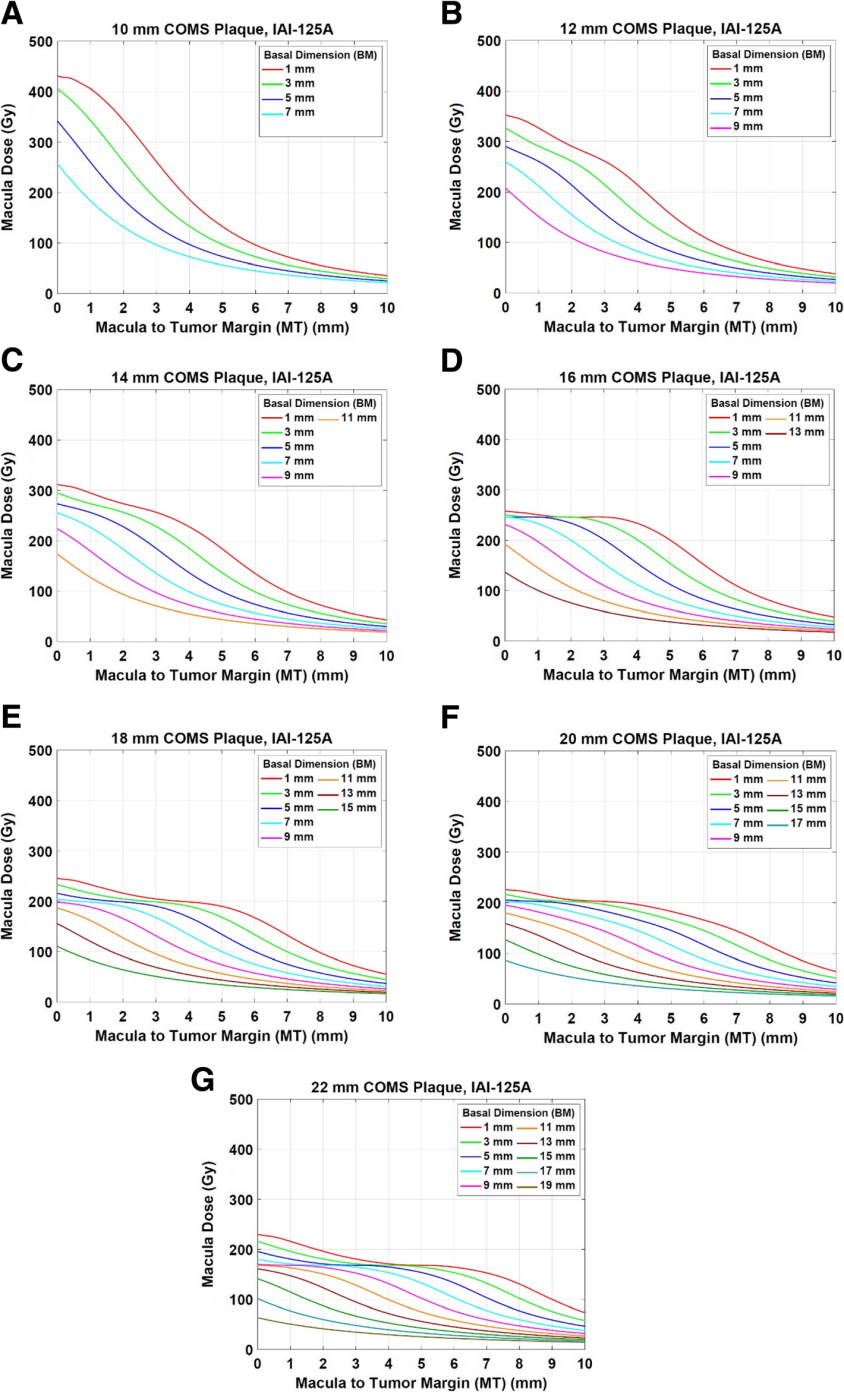
**a**-**g** Macula dose as a function of macula-to-tumor margin distance (MT) for various tumor basal dimensions (BM) for seven COMS plaques loaded with ^125^I seeds (model IAI-125A) when 85 Gy is prescribed to a central-axis depth of 5 mm

Optic disc dose and macula dose for standard COMS plaques loaded with ^125^I seeds (model IAI-125A) are shown in Figs. 2 and 3, respectively. Figure 2a-g presents optic disc dose as a function of DT up to 10 mm for various BDs in 2 mm intervals for seven COMS plaques when 85 Gy is prescribed at a central-axis depth of 5 mm for an irradiation time of 168 h. Optic disc dose decreases with increasing DT and increasing BD. For the plaques ≥16 mm, however, there exist some regions where optic disc dose does not change much with DT (i.e., flat regions in Fig. 2d-g). This usually occurs within short distances (≤5 mm of DT) when BD is less than or equal to 5 mm. For example, for the 16 mm COMS plaque (Fig. 2d), the graph for BD = 1 mm does not vary a lot at <3 mm of DT and the graph for BD = 3 mm does not change much at <2 mm of DT, respectively. Plaque size also determines optic disc dose and the shape of dose curves. Macula dose is displayed in Fig. 3a-g as a function of MT for various BMs. Similar patterns to optic disc dose are observed. Optic disc dose and macula dose for the other two seed models (2301 and I25.S16) are presented as Additional file 1 (data not shown here).

There are variations of optic disc dose and macula dose among seed models. The maximum relative differences (%) in optic disc dose between IAI-125A and 2301, between IAI-125A and I25.S16, and between 2301 and I25.S16 are 7.74, 5.89 and 5.28%, respectively. Corresponding maximum relative differences (%) for macula dose are 7.39, 5.64 and 5.28%.

### Dose conversion factors for different prescription depths

Dose conversion factors for different prescription depths from 1 mm to 10 mm in 1 mm intervals for standard COMS plaques loaded with ^125^I seed (model IAI-125A) are tabulated in Table 3. Based on the COMS protocols for tumors with apical height <5 mm [14], dose conversion factors were normalized to a depth of 5 mm. The factors increase with increasing prescription depth. The factors increase with increasing plaque size for a prescription depth <5 mm but the opposite is observed for a depth >5 mm. Table 3 is used for both optic disc dose and macula dose estimation when a prescription depth is not 5 mm. Dose conversion factors for the other two ^125^I seed models (2301 and I25.S16) are provided as Additional file 2 (data not shown here). The differences of the factors among seed models increase with increasing prescription depth and decreasing plaque size. Maximum absolute differences between IAI-125A and 2301, between IAI-125A and I25.S16, and between 2301 and I25.S16 are 0.08, 0.1 and 0.02, respectively.

**Table 3:** Dose conversion factors (ratios of total reference air kerma per seed) for different prescription depths (1 mm – 10 mm in 1 mm intervals) for standard COMS plaques loaded with ^125^I seeds (model IAI-125A). A reference depth for dose conversion factors is 5 mm

Prescription depth (mm)	Plaque size (mm) in diameter						
	10	12	14	16	18	20	22
1	0.29	0.33	0.37	0.42	0.44	0.47	0.47
2	0.41	0.45	0.48	0.52	0.55	0.58	0.59
3	0.57	0.60	0.63	0.66	0.68	0.70	0.71
4	0.77	0.78	0.80	0.81	0.83	0.84	0.85
5	1.00	1.00	1.00	1.00	1.00	1.00	1.00
6	1.27	1.25	1.23	1.22	1.20	1.18	1.18
7	1.59	1.54	1.50	1.47	1.43	1.40	1.38
8	1.95	1.88	1.82	1.76	1.69	1.64	1.61
9	2.37	2.27	2.18	2.10	2.00	1.92	1.87
10	2.84	2.71	2.59	2.48	2.34	2.24	2.17

### Estimation of optic disc dose without dose calculations: clinical application of this study

Optic disc dose (Fig. 2), macula dose (Fig. 3) and dose conversion factors (Table 3) presented in the current study can be conveniently used in clinic. As an example, there is a clinical case in which BD is 3 mm, DT is 3 mm and apical height is 4 mm. A clinician wants to prescribe 85 Gy to the tumor apex (i.e., 4 mm) using a 10 mm COMS plaque loaded with ^125^I seeds (model IAI-125A). From the data obtained in this study, optic disc dose is about 145 Gy for the 10 mm COMS plaque and for a prescription depth of 5 mm (Fig. 2a). The dose conversion factor for the 10 mm COMS plaque and for a prescription depth of 4 mm is 0.77 (Table 3). Thus, for this clinical case, expected optic disc dose is 112 Gy (=145 Gy × 0.77) which is lower than that when prescribed at 5 mm by 33 Gy. If tumor apex is higher (for instance, 6 mm) for the same tumor, optic disc dose for the same ^125^I seed model is about 184 Gy (=145 Gy × 1.27) (Table 3).

## Discussion

Our in-house brachytherapy dose calculation program demonstrated similar accuracy in brachytherapy dose calculations to commercial TPS. As presented in Tables 1 and 2, dose differences at central-axis and off-axis points between our in-house program and the three TPS used in Rivard et al.’s study were <2.4% except for the lacrimal gland point for plaque positions #1 and #3. Some seeds in plaque positions #1 and #3 have small polar angles (< 40 degrees) to the lacrimal gland point. At small polar angles, anisotropy function values vary more dramatically with polar angle than at large polar angles, leading to larger uncertainty in the interpolation of anisotropy function values. As mentioned in the Methods, Rivard et al.’s study used smaller step size over distance “r” and polar angle “θ” for anisotropy function than our in-house program (AAPM TG-43 U1), causing larger dose differences (~ 4.4%) between the two studies at the lacrimal gland point for plaque positions #1 and #3 than at the other dose points.

**Fig. 4:**
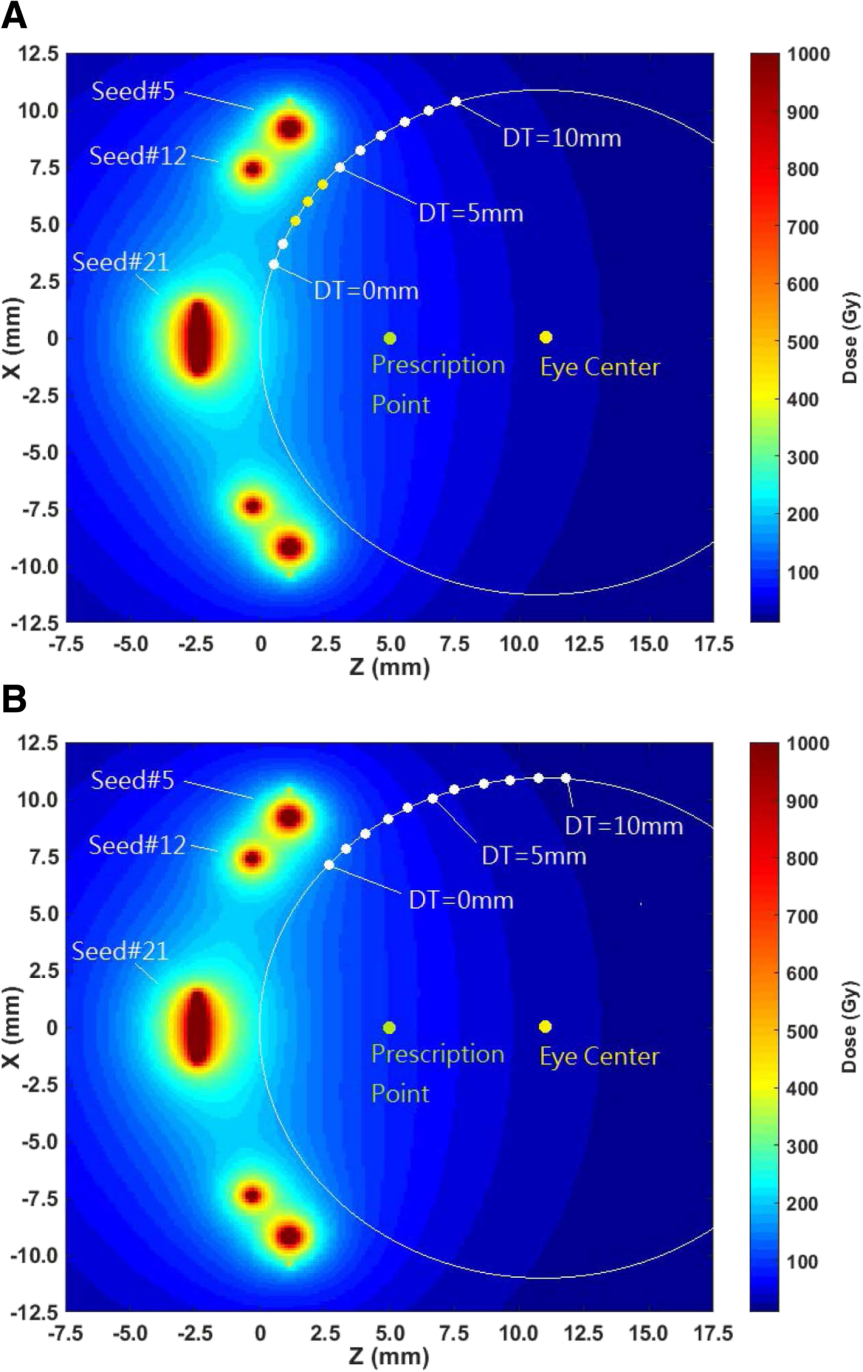
Optic disc dose clouds for a 22 mm COMS plaque loaded with ^125^I (model: IAI-125A) seeds for a tumor with **a** 5 mm basal dimension (BD) and **b** 13 mm basal dimension (BD). The positions in yellow (DT = 2 mm – 4 mm) in the **a** represent where dose does not change much

This study showed that optic disc dose and macula dose strongly depend on distance from tumor margin (DT and MT) and tumor basal dimension (BD and BM). In the COMS protocols, coordinates for the optic disc and macula are determined by the combination of these two parameters. Hence, the two parameters determine optic disc dose and macula dose. At close proximity (up to about 1 cm) of ^125^I seeds (plaque), the inverse square law effect is severe. At farther distances, however, the radial dose function for ^125^I seeds drastically decreases with distance because of its low photon energy (average energy: 28 keV) and consequently, rapid dose fall-off is observed. As a result, optic disc dose and macula dose decrease as DT and MT increase (Figs. 2 and 3), respectively. Optic disc dose and macula dose also decrease as BD and BM increase (Figs. 2 and 3), respectively because optic disc and macula become far away from the center of tumor as BD and BM increase, respectively. For plaques ≥16 mm, however, there are regions in which dose does not change much with distance (Figs. 2d-g and 3d-g). This occurs particularly for small basal dimensions at a tumor margin-to-critical structure distance ≤5 mm. As shown in Fig. 4a, for BD of 5 mm, as DT increases, an optic dose point becomes far away from seed #21 but simultaneously close to seeds #12 and #5. As a result, optic disc dose points between 2 mm and 4 mm of DT are in the same dose color map (three yellow dots in Fig. 4a) and optic disc dose is invariant within that region. On the other hand, for BD of 13 mm, an optic disc dose point becomes far away from seeds #12 and #5 with increasing DT. Therefore, each dose point between 2 mm and 4 mm is located in a different dose color map and optic disc dose decreases as DT increases (Fig. 4b). For the same reason, similar patterns are observed in macula dose (Fig. 3).

Optic disc dose and macula dose also have dependence on prescription depth and plaque size. The dependence can be explained with trends of dose conversion factors (Table 3) and TRAK values per seed (Table 4) as follows. First, for each plaque size, TRAK per seed to obtain a prescribed dose to a prescription depth increases with increasing prescription depth because a deeper prescription depth requires higher TRAK per seed. Hence, dose conversion factors increase with prescription depth. Second, for each prescription depth, TRAK per seed does not continuously decrease with increasing plaque size due to the number of seeds used in each COMS plaque (e.g., 24 seeds for 20 mm plaque and 21 seeds for 22 mm plaque). Thus, dose conversion factors do not always continuously increase (depth <5 mm) or decrease (depth >5 mm) with increasing plaque size. Third, TRAK per seed increases more rapidly with increasing prescription depth for smaller plaques than for larger plaques. The following example for seed model IAI-125A supports this trend. For the 10 mm plaque, TRAK per seed increases from 299.9 μGym^2^ to 2923.5 μGym^2^ (9.7-fold increase) when a prescription depth increases from 1 mm to 10 mm. On the other hand, for the 22 mm plaque, the increase of TRAK per seed by the depth increase from 1 mm to 10 mm is 4.6-fold (from 157.3 μGym^2^ to 718.6 μGym^2^). Thus, dose conversion factors increase more rapidly with increasing prescription depth for smaller plaques than for larger plaques, resulting in the increase in dose conversion factors with plaque size at a depth <5 mm but the decrease with plaque size at a depth >5 mm.

**Table 4:** TRAK per seed (in μGym^2^) for different prescription depths (1 mm – 10 mm in 1 mm intervals) for standard COMS plaques loaded with ^125^I seeds (model IAI-125A)

Prescription depth (mm)	Plaque size (mm) in diameter						
	10	12	14	16	18	20	22
1	299.9	226.3	161.2	191.6	134.8	132.3	157.3
2	424.9	306.2	212.2	241.0	168.0	162.0	194.2
3	587.2	406.6	274.4	301.3	206.3	195.8	234.6
4	788.9	530.2	349.5	373.9	251.2	234.7	280.1
5	1030.6	677.1	438.1	459.8	303.2	279.4	331.2
6	1311.2	848.0	540.9	559.7	363.2	330.7	389.3
7	1634.7	1045.2	659.3	675.4	432.6	389.9	455.7
8	2008.7	1274.1	797.3	810.4	513.4	458.6	532.3
9	2438.7	1536.9	955.4	965.1	605.8	537.1	619.8
10	2923.5	1833.6	1133.9	1140.1	710.4	626.0	718.6

There exist dose differences among seed models. The differences are caused by differences of the parameters used in the AAPM TG-43 U1 dosimetry formalism which result from different seed geometry and internal construction among three seed models [16, 19]. As reported in the Results, the differences can be fairly significant (up to 7.7%) and similar results were reported by Thomson et al. [19]. Thomson et al. performed MC calculations for a 16 mm COMS plaque loaded with ^125^I seeds under TG-43 assumptions (i.e., a homogeneous medium) and showed that doses differed by up to 11% for different seed models [19].

The results presented in this study will be beneficial to the clinic using ^125^I COMS plaques and help improve current clinical practice as follows. First, this study would allow clinicians to estimate optic disc dose and macula dose without dose calculations. Figures 2 and 3 and Table 3 or the data in additional files can be easily looked up as in the example discussed in the Results. In this study, the calculations were based on a prescribed dose of 85 Gy. In clinical cases where a prescribed dose is different from 85 Gy (e.g., re-irradiation or treatment of benign lesions), a dose scaling factor (prescribed dose (Gy)/85 Gy) can be multiplied by optic disc dose or macula dose obtained in this study to estimate dose to the critical structures. Second, if an estimated dose to the OARs is high enough to be paid attention to, clinicians may take an action to reduce dose to the OARs by prescribing to a different depth or by the use of a notched plaque [20]. As discussed in the example in the Results, prescribing 85 Gy to 4 mm (recent ABS guidelines [18]: prescribing at the tumor apex for all medium-sized choroidal melanomas) can give lower dose to the optic disc than prescribing 85 Gy to 5 mm (COMS protocols [14]: prescribing at 5 mm for the tumor apex <5 mm and to the apex for the tumor apex ≥5 mm [15]) (112 Gy vs. 145 Gy) due to lower TRAK per seed at a shallower depth. For the tumor apex ≥5 mm, prescribing dose to a shallower depth would reduce optic disc dose but tumor coverage can be compromised. Third, this study would enable clinicians to correlate clinical outcomes for vision with optic disc dose or macula dose. There have been no good published correlation data between clinical outcomes and radiation dose. If clinical outcomes for vision are available along with corresponding distance from tumor margin (DT and MT), tumor basal dimensions (BD and BM), plaque size, prescription depth, prescribed dose and seed model, one can correlate the outcomes with optic disc dose and macula dose which can be looked up from the results presented in this study. From the correlation data, clinicians can anticipate outcomes for vision for a given clinical situation and find a possible way to reduce dose to the critical structures before treatment. Furthermore, using the correlation data, tolerance dose to the optic disc or macula in ocular brachytherapy, which has not been known yet, can be investigated.

## Conclusions

This study has comprehensively examined optic disc dose and macula dose as a function of distance from tumor margin in ocular brachytherapy using ^125^I COMS plaques and has shown that dose to the critical structures has dependence on multiple parameters such as distance from tumor margin, tumor basal dimension, prescription depth, plaque size, and seed model. In any clinic or institution utilizing ^125^I COMS plaques, the dosimetry data provided in this study can be looked up to estimate optic disc dose and macula dose without dose calculations in a TPS.

## Additional files

Additional file 1: 4 figures: optic disc dose (1 for each seed model, 2301 and I25.S16) and macula dose (1 for each seed model, 2301 and I25.S16). (ZIP 5849 kb)

Additional file 2: 2 tables: dose conversion factors for two seed models (2301 and I25.S16). (ZIP 23 kb)

## Abbreviations

AAPM: American Association of Physicists in Medicine
ABS: American Brachytherapy Society
BD: Tumor basal dimension at center in the direction from optic disc
BM: Tumor basal dimension at center in the direction from macula
COMS: Collaborative Ocular Melanoma Study
DT: Distance from optic disc to tumor margin
MT: Distance from macula to tumor margin
OARs: Organs at risk
S_k_: Air-kerma strength
TG: Task group
TPS: Treatment planning system
TRAK: Total reference air kerma
