# Supplementary figures and images for: A practical approach to estimating optic disc dose and macula dose without treatment planning in ocular brachytherapy using 125I COMS plaques
Source: Radiat Oncol. 2018 Nov 13;13:221. doi: 10.1186/s13014-018-1166-z (PMC6234692; doi:10.1186/s13014-018-1166-z)

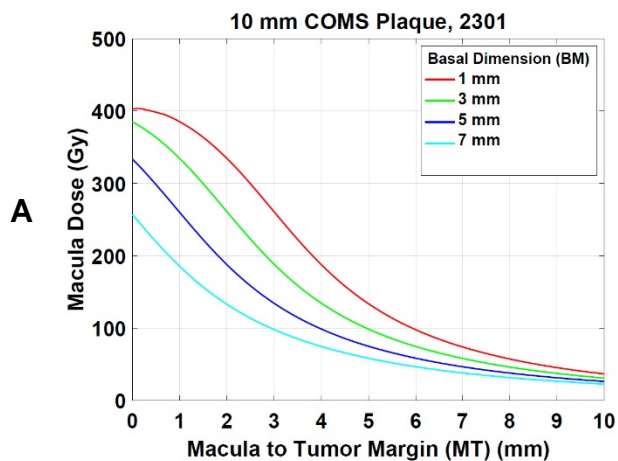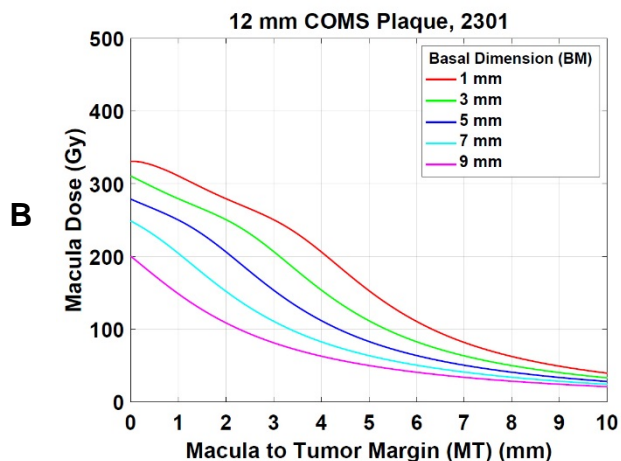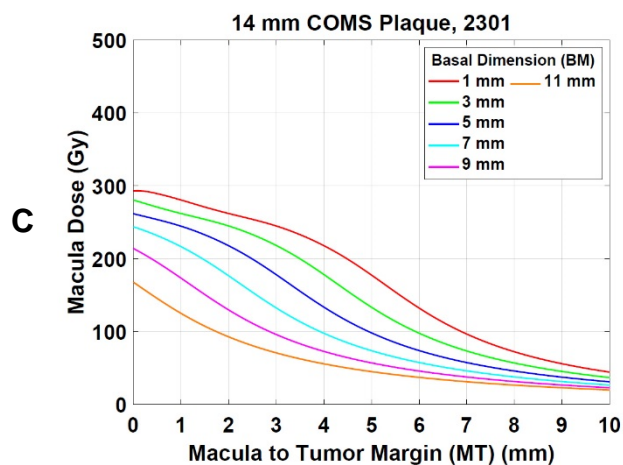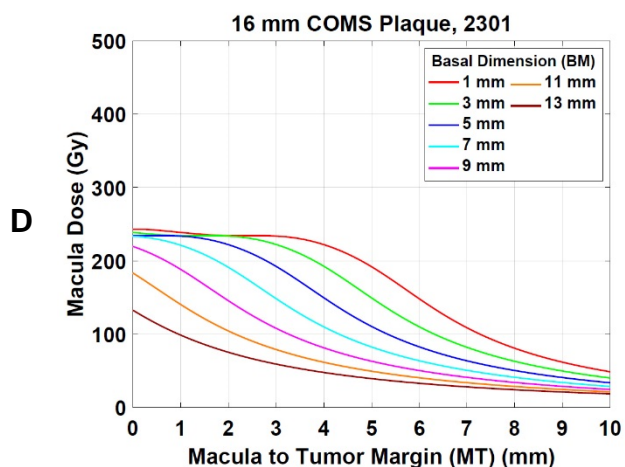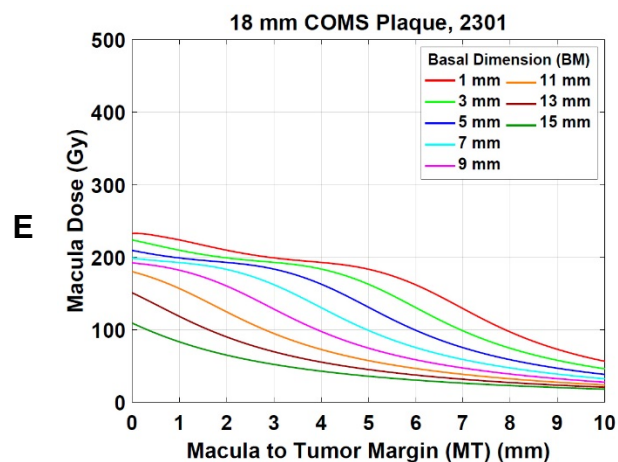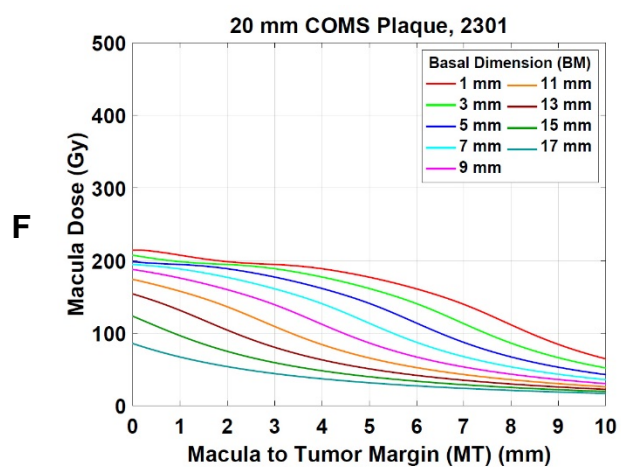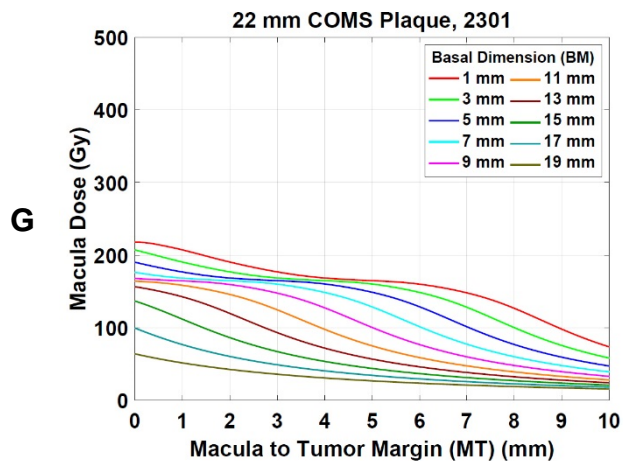

Supplement: Supplementary file 1 — 4 figures: optic disc dose (1 for each seed model, 2301 and I25.S16) and macula dose (1 for each seed model, 2301 and I25.S16). (ZIP 5849 kb) [file 13014_2018_1166_MOESM1_ESM.zip › Model_2301_Macula.pdf]

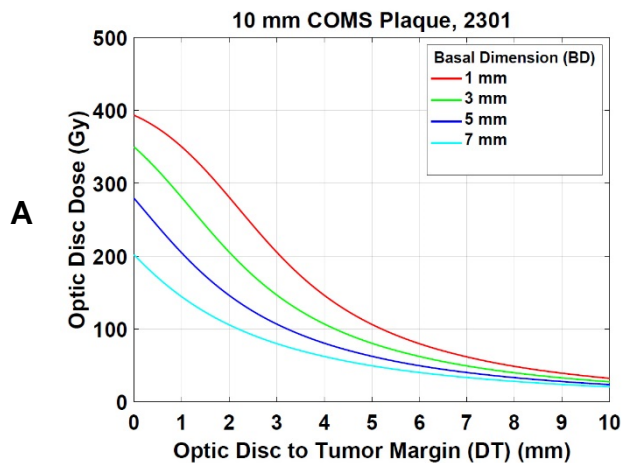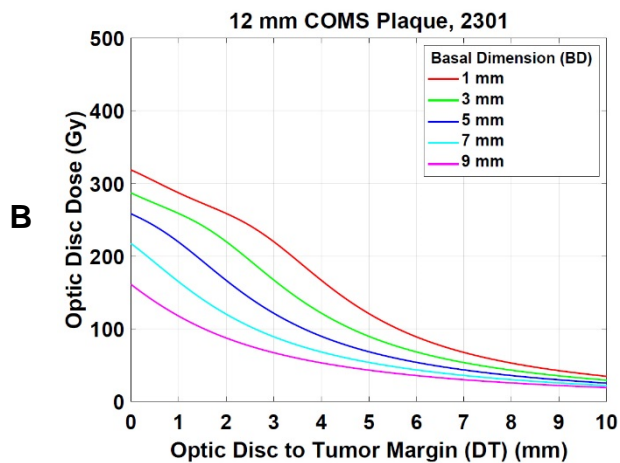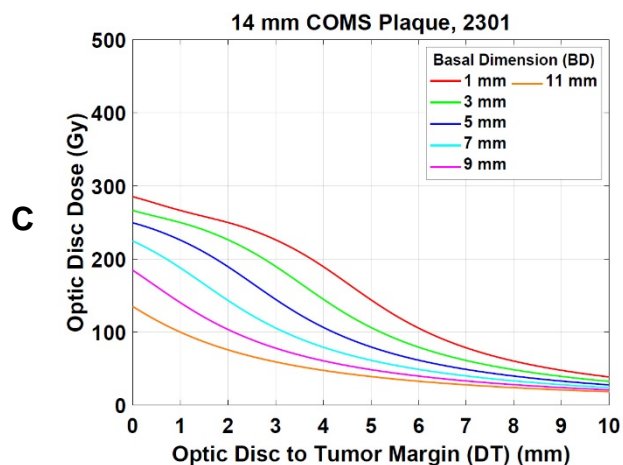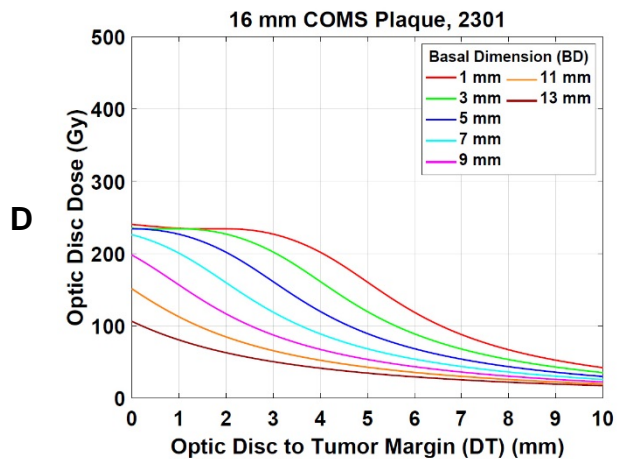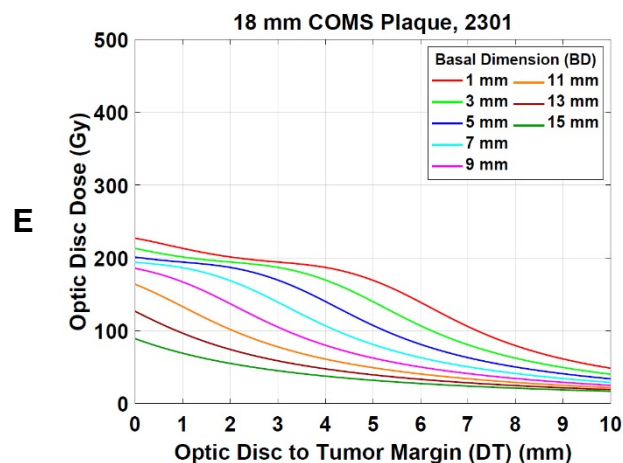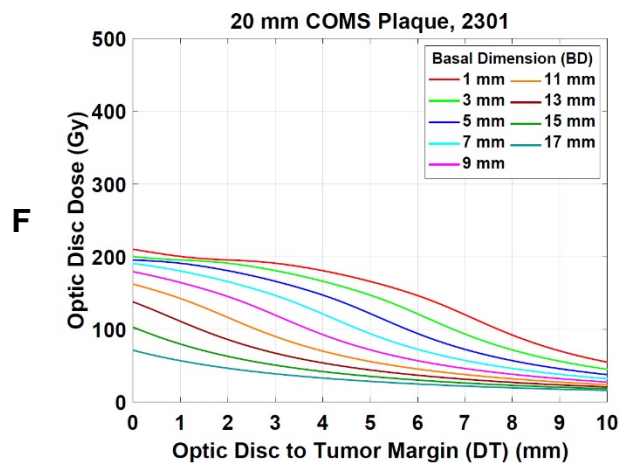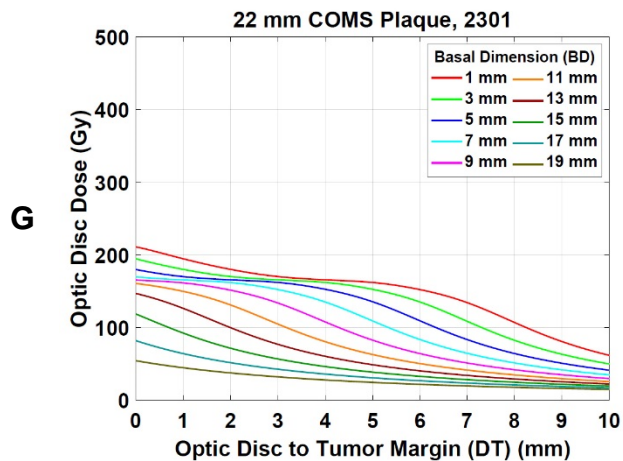

Supplement: Supplementary file 1 — 4 figures: optic disc dose (1 for each seed model, 2301 and I25.S16) and macula dose (1 for each seed model, 2301 and I25.S16). (ZIP 5849 kb) [file 13014_2018_1166_MOESM1_ESM.zip › Model_2301_OpticDisc.pdf]

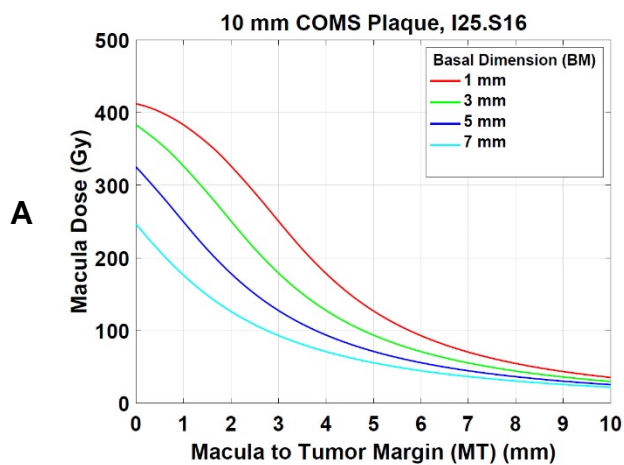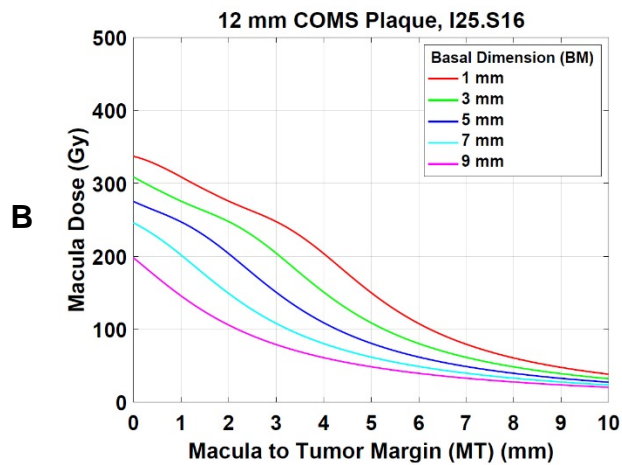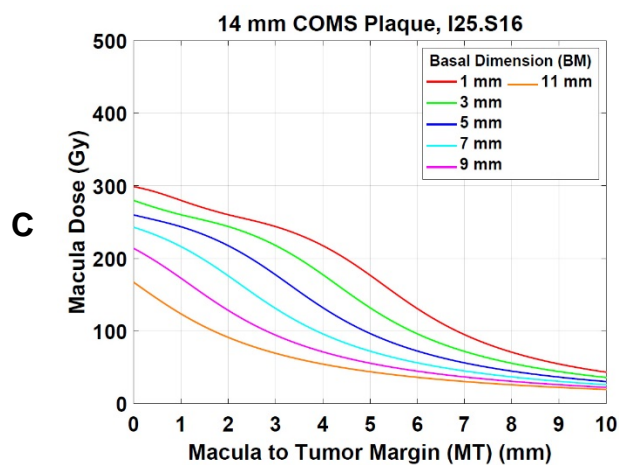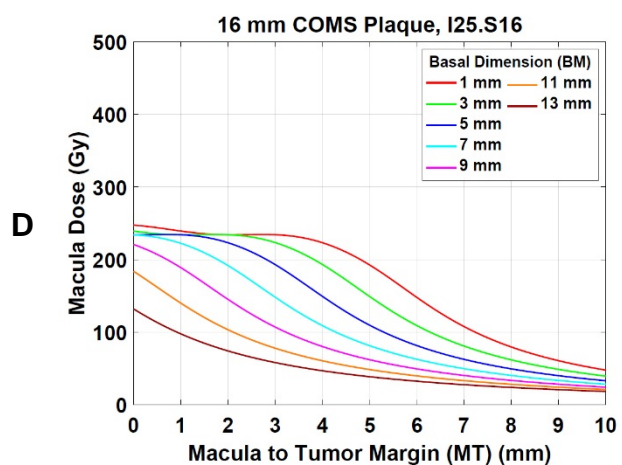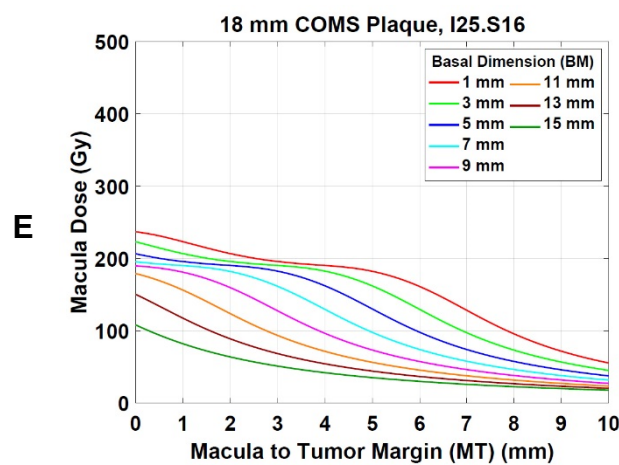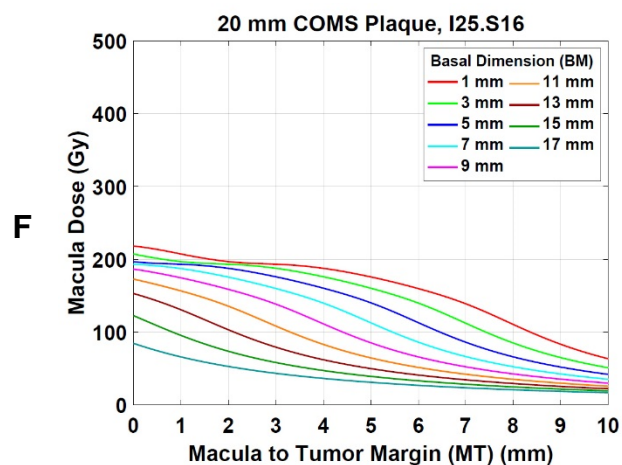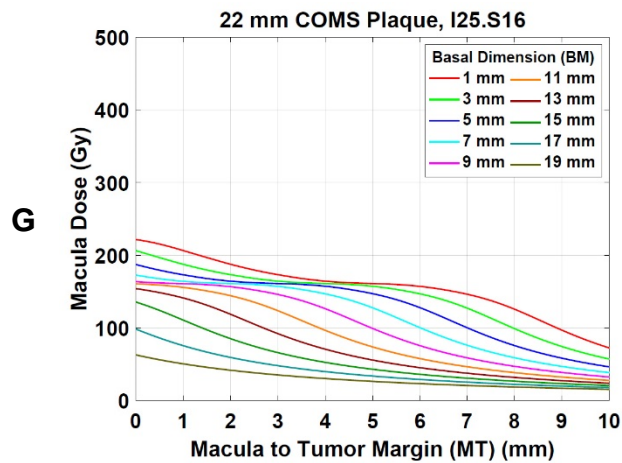

Supplement: Supplementary file 1 — 4 figures: optic disc dose (1 for each seed model, 2301 and I25.S16) and macula dose (1 for each seed model, 2301 and I25.S16). (ZIP 5849 kb) [file 13014_2018_1166_MOESM1_ESM.zip › Model_I25.S16_Macula.pdf]

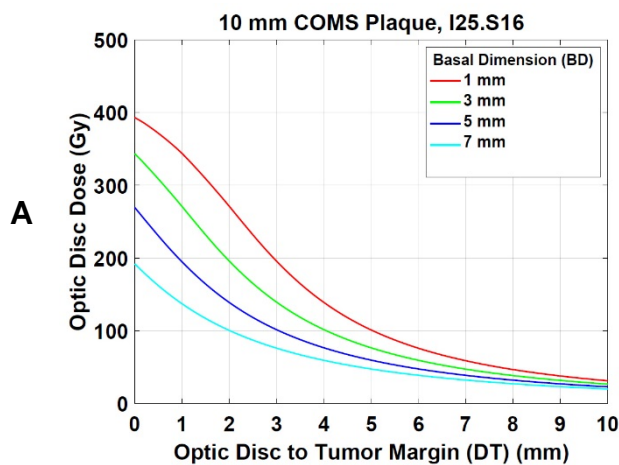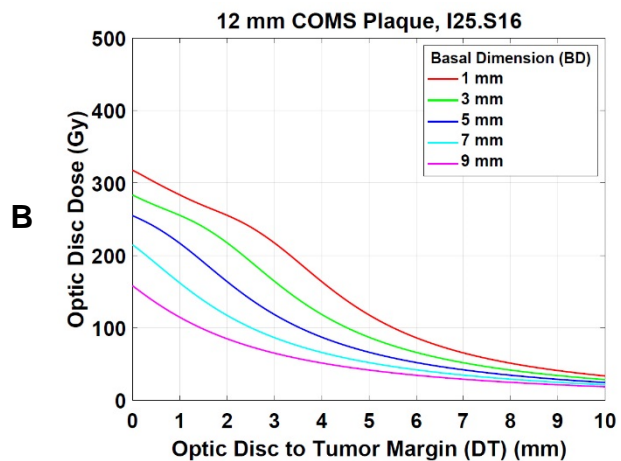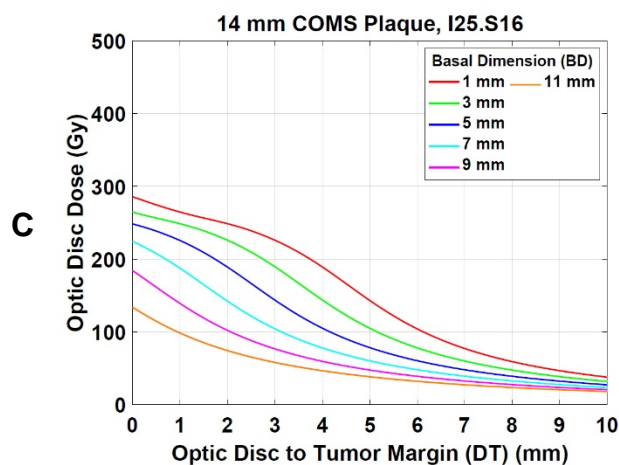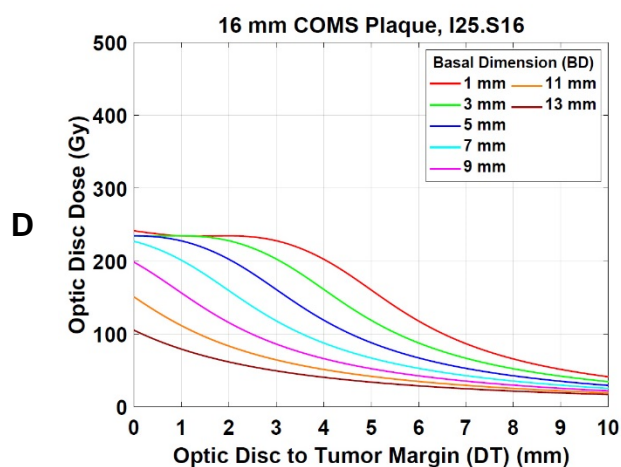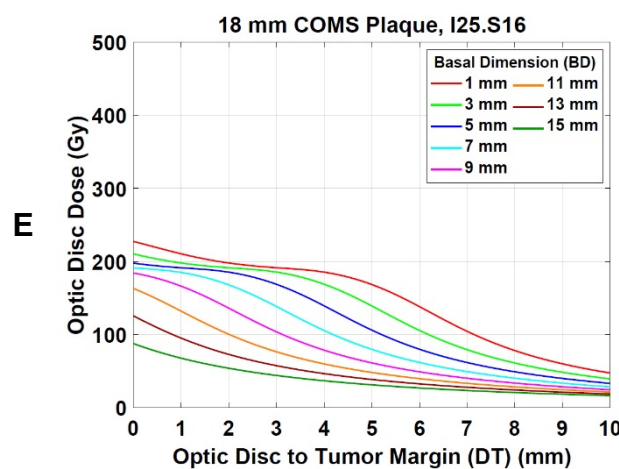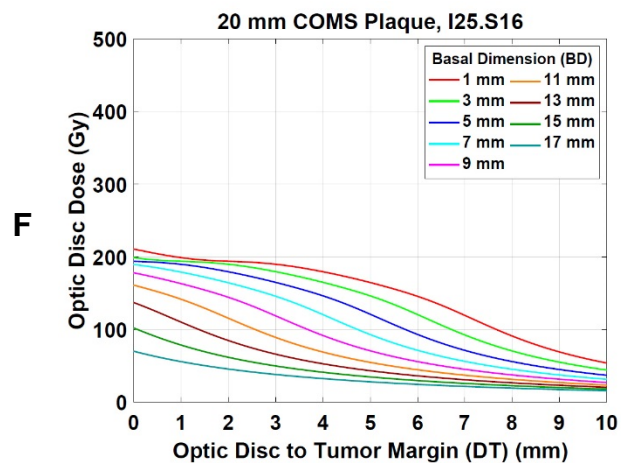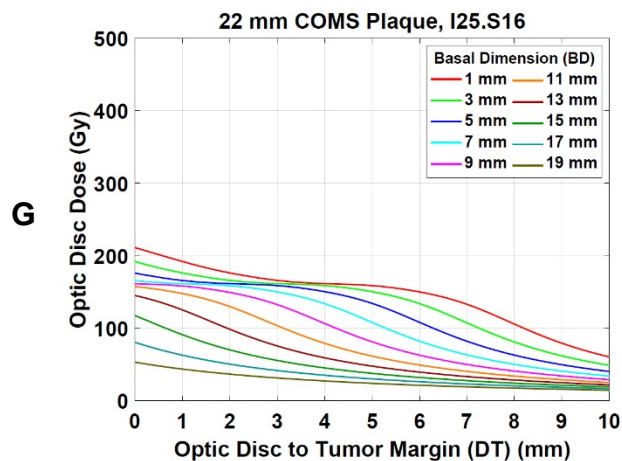

Supplement: Supplementary file 1 — 4 figures: optic disc dose (1 for each seed model, 2301 and I25.S16) and macula dose (1 for each seed model, 2301 and I25.S16). (ZIP 5849 kb) [file 13014_2018_1166_MOESM1_ESM.zip › Model_I25.S16_OpticDisc.pdf]
