# Supplementary material for: A practical approach to estimating optic disc dose and macula dose without treatment planning in ocular brachytherapy using 125I COMS plaques
Source: Radiat Oncol. 2018 Nov 13;13:221. doi: 10.1186/s13014-018-1166-z (PMC6234692; doi:10.1186/s13014-018-1166-z)
Supplement: Supplementary file 2 — 2 tables: dose conversion factors for two seed models (2301 and I25.S16). (ZIP 23 kb) [file 13014_2018_1166_MOESM2_ESM.zip › DoseConversionFactor125I_Model_2301_Re.docx]

**Table.** Dose conversion factors (ratios of total reference air kerma per seed) for different prescription depths (1 mm – 10 mm in 1 mm intervals) for standard COMS plaques loaded with ^125^I (model 2301) seeds. A reference depth for dose conversion factors is 5 mm.

| Prescription depth (mm) | Plaque size (mm) in diameter | | | | | | |
| --- | --- | --- | --- | --- | --- | --- | --- |
|  | **10** | **12** | **14** | **16** | **18** | **20** | **22** |
| 1 | 0.31 | 0.35 | 0.39 | 0.44 | 0.46 | 0.49 | 0.49 |
| 2 | 0.43 | 0.47 | 0.50 | 0.54 | 0.57 | 0.60 | 0.60 |
| 3 | 0.59 | 0.62 | 0.64 | 0.67 | 0.69 | 0.71 | 0.72 |
| 4 | 0.78 | 0.79 | 0.81 | 0.82 | 0.83 | 0.85 | 0.85 |
| 5 | 1.00 | 1.00 | 1.00 | 1.00 | 1.00 | 1.00 | 1.00 |
| 6 | 1.27 | 1.25 | 1.23 | 1.21 | 1.19 | 1.18 | 1.17 |
| 7 | 1.57 | 1.53 | 1.50 | 1.46 | 1.42 | 1.39 | 1.37 |
| 8 | 1.92 | 1.86 | 1.80 | 1.74 | 1.67 | 1.62 | 1.59 |
| 9 | 2.32 | 2.22 | 2.14 | 2.06 | 1.96 | 1.89 | 1.84 |
| 10 | 2.76 | 2.63 | 2.52 | 2.41 | 2.28 | 2.18 | 2.11 |
